# Association Between Aflatoxin B1 Albumin Adduct Levels and Tuberculosis Infection Among HIV+ Ghanaians.

**Published:** 2011

**Authors:** John Keenan, Pauline Jolly, Peter Preko, Joseph Baidoo, Jia-Sheng Wang, Timothy D. Phillips, Jonathan H. Williams, Gerald McGwin

**Affiliations:** 1 University of Alabama at Birmingham, Birmingham, Alabama, School of Public Health,; 2 St. Markus Hospital, Kumasi, Ghana,; 3 Department of Environmental Health Science, University of Georgia, Athens, Georgia,; 4 Department of Veterinary Integrative Biosciences, Texas A & M University, College Station, Texas,; 5 College of Agricultural and Environmental Sciences, University of Georgia, Griffin, GA.

**Keywords:** Aflatoxin, tuberculosis, HIV

## Abstract

**Background:**

Aflatoxin exposure has been shown to cause cell-mediated immune suppression and enhance HIV viral replication. Such immune suppression from aflatoxin can impair resistance to both infectious diseases and chronic infections.

**Methods:**

Hazard ratios (HRs) with 95% confidence intervals (CI) and a test for trend for opportunistic infections OI) among 141 HIV positive Ghanaians based on quartiles of aflatoxin B1 albumin adduct levels (AF-ALB) were calculated.

**Findings:**

HRs were significantly higher for developing symptomatic TB (HR 3.30, 95% CI 1.34–8.11) for those in the highest AF-ALB quartile compared to the lowest. Significantly higher HRs were not observed for other infections investigated.

**Conclusions:**

Those with the highest levels AF-ALB from dietary intake have an increased hazard of symptomatic TB but not malaria, HBV, or pneumonia.

## Introduction

The immunosuppressive effects of HIV and the increased risk of opportunistic infections (OIs) are common knowledge. Despite the decline of morbidity and mortality from OIs with highly active antiretroviral therapy treatment among HIV positive individuals in developed nations, OIs continue to cause significant morbidity and mortality in developing countries [1]. Similar to the deleterious, immune suppressive effects of HIV, aflatoxin exposure has been shown to cause immune suppression, particularly suppression of cell-mediated immune responses, in both human [2, 3] and animal studies [4–6]. Specifically, aflatoxin has been reported to decrease T and B lymphocyte activity [7], alter macrophage and neutrophil function [8–10], and diminish natural killer cell-mediated cytolysis [11]. Such immune suppression from aflatoxin has been shown to impair resistance to both infectious diseases and chronic infections [12]. Aflatoxins are produced from strains of fungi growing on crops such as maize, groundnuts and oilseeds [13]. These are important food sources in Ghana and have been well documented in the scientific literature as containing elevated aflatoxin levels. In 1978, a survey found that between 50–80% of groundnuts purchased in Ghana contained levels of aflatoxin in excess of FAO/WHO/UNICEF recommended levels for human consumption [14]. More recent studies have identified widespread maize aflatoxin contamination in both the percent of kernels contaminated [15] and the levels of aflatoxin identified [16]. Food prepared from aflatoxin contaminated raw materials also contributes to human risk. Fermented dough and kenkey balls (boiled maize balls wrapped in cornhusks) purchased in Ghana have each been reported to contain dramatically elevated levels of aflatoxin [17]. Aflatoxin B1 albumin adduct level (AF-ALB) is a common biomarker for dietary aflatoxin exposure [18] and is suggestive of exposure over a two to three month period [19].

While some authors have suggested a synergistic immune suppressive effect of HIV and aflatoxin exposure [20, 21], very little research is available on the joint effects. Previous, research reported that HIV positive individuals with high AF-ALB levels had lower levels of CD4+ T regulatory cells, lower levels of naive CD4+ T cells, reduced percentage of B-cells, and lower perforin expression on CD8+T-cells than those with low AF-ALB levels [3]. Specific to HIV progression is the role of aflatoxin in increasing HIV viral load. Both oxidative stress [22] and selenium deficiency [23] caused by aflatoxin exposure have been associated with increased HIV viral replication.

Protection from tuberculosis (TB) relies on both CD4+ and CD8+ T cells with the former described as the most important [24]. The role of CD4+T cell depletion promoting TB infection in mouse models has been demonstrated [25]. Further, the effects of reduced CD4+ T cells in HIV positive individuals greatly increases the susceptibility to incident infection and TB reactivation [26]. A recent study concluded that animal protection from TB requires *Mycobacterium tuberculosis* specific CD8+ T cells containing perforin [27].

The objective of this study was to investigate the effects of aflatoxin exposure on symptomatic OIs in a cohort of HIV positive individuals.

## Methods

This was a prospective, longitudinal, cohort study of 141 HIV positive Ghanaians, exposed to aflatoxin in their diet, investigating symptomatic, secondary disease development and/or progression. The participants were patients receiving care at St. Markus Hospital in Kumasi between February 2002 and December 2004. Participants voluntarily sought care at the clinic for their health needs. At baseline, participants were asked to complete a survey on socio-demographic characteristics. A 20mL blood sample was collected from each participant in EDTA vacutainer tubes and plasma samples were used for detection of AF-ALB and viral load. At each visit the attending physician recorded a maximum of four symptomatic diseases in the patient records. The five most frequent diseases were chosen for outcomes. Approval for the study was obtained from the Institutional Review Board of the University of Alabama at Birmingham (UAB) and the Committee on Human Research, Publication and Ethics, Kwame Nkrumah University of Science and Technology (KNUST) College of Health Sciences, Kumasi, Ghana.

## Laboratory analysis for AF-ALB, CD4+ T cell count and HIV viral load

Aflatoxin B1 albumin adduct levels (AF-ALB) levels in plasma were determined by radioimmunoassay (RIA) as previously described by Wang and colleagues [19]. Values were expressed as pmol AF-ALB/mg albumin. The detection limit of the assay was 0.01 pmol/mg albumin. The absolute CD4 count was a calculated product of the total lymphocyte count and the percentage of lymphocytes that were CD4+ T cells determined by BD LSR II Flow Cytometry (Becton Dickenson, Franklin Lakes, NJ). Absolute lymphocyte counts were derived from the white blood cell (WBO counts and leukocyte differential counts which were performed in the laboratory of the Department of Biochemistry at KNUST. Circulating CD4+ T cell populations were determined by flow cytometry using fluorescein FITC-labeled monoclonal antibody against CD4 (BD PharMingen, San Diego, CA). Isotype-matched controls (BD PharMingen) were used in all experiments. HIV RNA was measured using a quantitative reverse transcriptase polymerase chain reaction assay (Amplicor Monitor, Roche Diagnostic System, Brandersburg, NJ) and results were reported as HIV RNA copies/mL as reported previously [3]. All undetectable values (below 400 copies) were assigned a value of 399.

## Statistical Analysis

AF-ALB was treated as a categorical variable with patients divided into quartiles based on baseline AF-ALB levels. Age, follow-up time, baseline CD4 count, and HIV viral load were treated as continuous variables and compared across AF-ALB quartiles by ANOVA, while sex was treated as categorical compared using a chi-square test. A Cox proportional hazards model was used to calculate hazard ratios (HRs) and 95% confidence intervals (CIs) using the lowest AF-ALB level as the reference category. When repeated events were encountered the Cox model was modified to handle repeated events with The Prentice, Williams and Petersen conditional probability (PWP-CP) method. This method uses a counting process style with each event assigned to separate strata per participant [28]. Hazard ratios were calculated for tuberculosis, malaria, hepatitis, and pneumonia. The proportional hazards assumption was verified visually through a plot of the Cox-Snell residuals. Since some patients were lost to follow-up while others were censored when the study ended we experienced a combination of random and Type I, right censoring. Event time was recorded at time of diagnosis rather than time when disease began, thereby avoiding interval censoring. Separate analyses were conducted for each outcome and participants with prevalent disease were eliminated from the respective analysis. To investigate how each increasing AF-ALB exposure levels affected each disease, we conducted a test for trend. An additional analysis was conducted to investigate if AF-ALB levels influence the total number of reported diseases. A negative binomial regression was used to compare the incidence rate ratio for the development of any reported diseases for each participant.

## Results

Each of the 141 participants had detectable AF-ALB levels with a median value of 0.94 pmoL/mg albumin, received an average of 7.2 (SD 5.5, median 6.0) symptomatic disease diagnoses, returned for an average of 3.77 (SD 2.6, median 3.0) physician visits and were followed an average of 274.10 days (SD 256.9, median 208.0). The average time between visits was 90 days with a minimum of 33 days. The most commonly diagnosed diseases were malaria, herpes, tuberculosis, pneumonia, and hepatitis. The percentages receiving at least one diagnosis of each of the five diseases, including baseline diagnosis, were 48.9% malaria, 31.9% herpes, 31.2% tuberculosis, 20.6% hepatitis B, and 10.6% pneumonia. Of those diagnosed with at least one episode of herpes, 28.4% were prevalent cases, of which there were very few subsequent occurrences; therefore, HRs were not calculated for this disease. Demographic characteristics of the participants by AF-ALB quartile are displayed in Table 1. Univariate and multivariate analysis hazard ratios are displayed in Table 2. As none of the participants developing hepatitis B acquired a second, different hepatitis virus, hepatitis was treated as a single event and there was no increase in hazard of developing hepatitis for increased AF-ALB levels. While those with elevated AF-ALB levels had increased HRs for pneumonia, these findings were not significant. Tuberculosis was the only disease with a significant finding with those in the highest quartile having an HR of 3.39 (95% CI 1.15–9.98) compared to those in the lowest quartile. As with the HRs, the test for trend was only significant for TB. Comparison of an all disease incidence rates between the AF-ALB quartiles revealed no significant differences in incidence rates (data not shown).

## Discussion

The results of the current study demonstrated, for the first time an association between aflatoxin levels and an increased hazard for developing TB in HIV positive individuals. Significantly elevated HRs were not observed for the other diseases studied. Likewise, evaluation of all-disease incidence rates showed no difference for increased AF-ALB levels. This, all-disease incidence rate included the four diseases studied independently as well as other diseases such as lower and upper respiratory tract infections, candida, helminthiasis, anemia, and depression, to name a few.

Perhaps no other disease has been linked with HIV to the extent as TB and the effect TB has on HIV cannot be overstated. In 2009, among HIV positive individuals, there was an estimated 1.0–1.2 million incident cases of TB resulting in 0.4 million deaths from incident TB [26]. The immune suppression as a result of HIV alters the clinical course of TB and vice versa. In this study those with the highest levels of AF-ALB were at greater risk of developing symptomatic TB than those with the lowest levels. While it cannot be determined if these symptomatic episodes were from the same TB infection, a recent study determined that two-thirds of TB infections in HIV positive individuals in South Africa were a result of new infections [29]. There were elevated but non-significant HRs for elevated AF-ALB exposure and pneumonia and slightly diminished HRs for both malaria and hepatitis B. One cannot discern if the altered but non-significant HRs are due to limited sample size or actually due to no association. The various diseases each have specific immune responses, some of which may not be influenced by the particular immune suppressive effects of aflatoxin exposure. Further research is required to investigate these questions.

The results of this study may have been affected by several limitations. The power to detect significance may have been diminished due to the relatively small sample size. Also, some participants were censored early on, thereby reducing the possibility of the physicians recording symptomatic disease. Dividing the participants into quartiles resulted in fewer than 36 participants in each AF-ALB group. However, similar results were observed when participants were divided into two or three groups based on medians and tertiles, as were observed with the quartile division. Disease status was derived from medical records based on physician findings regardless of the means of obtaining the diagnosis; this may have resulted in misclassification of disease. However, such a misclassification would be independent of the exposure variable and would be considered non-differential misclassification. Participants were on a variety of medications including anti-retrovirals (ARVs). These medications were only targeted at either HIV or OI and not for aflatoxin treatment. Therefore, such medications would not be considered potential confounders. With particular respect to ARVs, limited research is available regarding the influence of ARVs on AF-ALB levels; some ARVs increase and others decrease cytochrome P450 (CYP450) enzymes that metabolize aflatoxin [30]. In our study 31.9% of participants were receiving one of two ARV regimens and the frequencies of those on ARVs did not differ between AF-ALB quartiles (p=0.67). Due to the similar number of participants on ARVs in each quartile, the limited evidence of the association between ARVs and AF-ALB, and the limited number of ARV regimens used in the study population, ARV use was not considered a potential confounder and was not adjusted for in the full multivariable model. Lastly, due to various factors including limited sample size, low frequencies of other OIs, and the strength of scientific literature regarding the effect on cell mediated immunity by dietary exposure to aflatoxin, we chose to include only those diseases most frequently seen in the population and those likely to require a cell-mediated immune response.

## Conclusion

Highest levels of AF-ALB appear to increase the hazard of OI symptomatic TB but not malaria, HBV, or pneumonia. There was a significant trend toward higher HRs with increased AF-ALB quartile. This increased hazard represents an important addition to the body of knowledge on the harmful effects of dietary aflatoxin exposure in individuals with HIV. As this study is the first to report an increased hazard of symptomatic TB in HIV positive individuals, additional research is warranted to further elucidate this relationship.

## Figures and Tables

**TABLE 1.** Demographic characteristics of total study participants by quartile of AF-ALB levels.

	AF-ALB Quartile 1 0.093>0.685* (n = 35)	AF-ALB Quartile 2 0.685≥ 0.939* (n = 35)	AF-ALB Quartile 3 0.939 ≥ 1.407* (n = 36)	AF-ALB Quartile 4 1.407 ≥ 3.48* (n = 35)	p-value
Age mean (SD)	39.00 (8.59)	36.58 (8.56)	39.54 (11.86)	39.69 (7.28)	0.47
Sex	12M/23F	11M/24F	15M/21F	12M/23F	0.83
Mean follow-up time in days (SD)	320 (250.72)	216 (216.45)	294 (283.52)	264 (270.60)	0.37
Number on ARV’s (%)	13 (37.14)	9 (25.71)	13 (36.11)	10 (28.57)	0.67
Mean baseline CD4 count (SD)	215.09 (130.89)	334.89 (287.42)	271.46 (178.82)	363.02 (327.24)	0.08
Mean baseline viral load (SD)	83,961.54 (240055.52)	53,341.09 (100105.51)	51,976.03 (129095.86)	137,852.63 (324368.25)	0.71

* AF-ALB levels are in pmol/mg albumin

**TABLE 2.** Univariate and multivariable hazard ratios for symptomatic disease by AF-ALB quartile.

Tuberculosis	Unadjusted analysis			Adjusted analysis#			Test for trend#
	Hazard Ratio	95% CI	p value	Hazard Ratio	95% CI	p value	p value
AF-ALB Quartile 1	Ref	---	---	Ref	---	---	0.03
AF-ALB Quartile 2	0.63	0.16–2.51	0.51	0.43	0.06–2.74	0.34	
AF-ALB Quartile 3	1.40	0.56–3.48	0.47	1.44	0.52–3.47	0.54	
AF-ALB Quartile 4	2.55	0.99–6.58	0.05	3.39	1.34–8.11	0.01	
Hepatitis BΨ							0.5
AF-ALB Quartile 1	Ref	---	---	Ref	---	---	
AF-ALB Quartile 2	0.90	0.15–5.41	0.91	1.11	0.17–6.36	0.97	
AF-ALB Quartile 3	0.41	0.04–3.92	0.44	0.36	0.06–5.22	0.59	
AF-ALB Quartile 4	0.47	0.05–4.53	0.51	0.37	0.04–7.03	0.51	
Malaria							
AF-ALB Quartile 1	Ref	----	---	Ref	----	---	0.42
AF-ALB Quartile 2	1.12	0.52–2.42	0.78	0.98	0.47–2.04	0.96	
AF-ALB Quartile 3	1.05	0.62–1.76	0.87	0.98	0.57–1.68	0.94	
AF-ALB Quartile 4	0.95	0.54–1.66	0.85	0.70	0.34–1.49	0.71	
Pneumonia							0.11
AF-ALB Quartile 1	Ref	----	---	Ref	----	---	
AF-ALB Quartile 2	1.12	0.21–6.08	0.89	0.53	0.06–5.92	0.68	
AF-ALB Quartile 3	2.40	0.73–7.90	0.15	2.50	0.77–9.21	0.12	
AF-ALB Quartile 4	1.68	0.41–6.79	0.47	1.59	0.48–9.07	0.33	

AF-ALB Quartile 1 0.093>0.685 pmol/mg albumin, AF-ALB Quartile 2 0.685≥ 0.939 pmol/mg albumin, AF-ALB Quartile 3 0.939 ≥ 1.407 pmol/mg albumin, AF-ALB Quartile 3 1.407 ≥ 3.48 pmol/mg albumin

# Adjusted for HIV viral load, and CD4 count

Ψ Hepatitis B analysis was treated as single even

## References

[1] Kaplan JE, Benson C, Holmes KH, Brooks JT, Pau A, Masur H (2009) Guidelines for prevention and treatment of opportunistic infections in HIV-infected adults and adolescents: recommendations from CDC, the National Institutes of Health, and the HIV Medicine Association of the Infectious Diseases Society of America. MMWR Recomm Rep. 58: 1–207; quiz CE201–204. 19357635

[2] Jiang Y, Jolly PE, Ellis WO, Wang JS, Phillips TD, Williams JH (2005) Aflatoxin B1 albumin adduct levels and cellular immune status in Ghanaians. Int Immunol. 17: 807–814. 15944194 10.1093/intimm/dxh262

[3] Jiang Y, Jolly PE, Preko P, Wang JS, Ellis WO, Phillips TD, Williams JH (2008) Aflatoxin-related immune dysfunction in health and in human immunodeficiency virus disease. Clin Dev Immunol. 2008: 790309. 18695741 10.1155/2008/790309 PMC2496958

[4] Bondy GS, Pestka JJ (2000) Immunomodulation by fungal toxins. J Toxicol Environ Health B Crit Rev. 3: 109–143. 10834078 10.1080/109374000281113

[5] Ghosh RC, Chauhan HV, Jha GJ (1991) Suppression of cell-mediated immunity by purified aflatoxin B1 in broiler chicks. Vet Immunol Immunopathol. 28: 165–172. 1907777 10.1016/0165-2427(91)90138-3

[6] Neiger RD, Johnson TJ, Hurley DJ, Higgins KF, Rottinghaus GE, Stahr H (1994) The short-term effect of low concentrations of dietary aflatoxin and T-2 toxin on mallard ducklings. Avian Dis. 38: 738–743. 7702506

[7] Reddy RV, Taylor MJ, Sharma RP (1987) Studies of immune function of CD-1 mice exposed to aflatoxin B1. Toxicology. 43: 123–132. 2433813 10.1016/0300-483x(87)90002-3

[8] Cusumano V, Rossano F, Merendino RA, Arena A, Costa GB, Mancuso G, Baroni A, Losi E (1996) Immunobiological activities of mould products: functional impairment of human monocytes exposed to aflatoxin B1. Res Microbial. 147: 385–391. 10.1016/0923-2508(96)84713-9 8763624

[9] Neldon-Ortiz DL, Qureshi MA (1992) Effects of AFB1 embryonic exposure on chicken mononuclear phagocytic cell functions. Dev Comp Immunol. 16: 187–196. 1499838 10.1016/0145-305x(92)90018-8

[10] Moon EY, Rhee OK, Pyo S (1999) In vitro suppressive effect of aflatoxin B1 on murine peritoneal macrophage functions. Toxicology. 133: 171–179. 10378483 10.1016/s0300-483x(99)00023-2

[11] Reddy RV, Sharma RP (1989) Effects of aflatoxin B1 on murine lymphocytic functions. Toxicology. 54: 31–44. 2492685 10.1016/0300-483x(89)90076-0

[12] Williams JH, Phillips TD, Jolly PE, Stiles JK, Jolly CM, Aggarwal D (2004) Human aflatoxicosis in developing countries: a review of toxicology, exposure, potential health consequences, and interventions. Am J Clin Nutr. 80: 1106–1122. 15531656 10.1093/ajcn/80.5.1106

[13] Gourama H, Bullerman L (1995) *Aspergillus flavus* and *Aspergillus parasiticus,* aflatoxigenic fungi of concern in foods and feed—a review. J Food Prot. 58:10.4315/0362-028X-58.12.1395 31159052

[14] Mintah S, Hunter RB (1978) The incidence of aflatoxin found in groundnuts (*Arachis hypogea L*.) purchased from markets in and around Accra, Ghana. Peanut Sci. 5: 13–16.

[15] Awuah RT, Kpodo KA (1996) High incidence of *Aspergillus flavus* and aflatoxins in stored groundnut in Ghana and the use of a microbial assay to assess the inhibitory effects of plant extracts on aflatoxin synthesis. Mycopathologia. 134: 109–114. 8981776 10.1007/BF00436873

[16] Kpodo K, Thrane U, Hald B (2000) Fusaria and fumonisins in maize from Ghana and their co-occurrence with aflatoxins. Int J Food Microbial. 61: 147–157. 10.1016/s0168-1605(00)00370-6 11078165

[17] Jespersen L, Halm M, Kpodo K, Jakobsen M (1994) Significance of yeasts and moulds occurring in maize dough fermentation for ‘kenkey’ production. Int J Food Microbial. 24: 239–248. 10.1016/0168-1605(94)90122-8 7703017

[18] Montesano R, Hainaut P, Wild CP (1997) Hepatocellular carcinoma: from gene to public health. J Natl Cancer Inst. 89: 1844–1851. 9414172 10.1093/jnci/89.24.1844

[19] Wang JS, Qian GS, Zarba A, He X, Zhu YR, Zhang BC, Jacobson L, Gange SJ, Munoz A, Kensler TW, (1996) Temporal patterns of aflatoxin-albumin adducts in hepatitis B surface antigen-positive and antigen-negative residents of Daxin, Qidong County, People’s Republic of China. Cancer Epidemiol Biomarkers Prev. 5: 253–261. 8722216

[20] Mlombe Y, Dzamalala C, Chisi J, Othieno-Abinya N (2009) Oesophageal cancer and Kaposi’s sarcoma in Malawi: a comparative analysis. Malawi Med J. 21: 66–68. 20345007 10.4314/mmj.v21i2.44562 PMC3345737

[21] Hendrickse RG, Maxwell SM, Young R (1989) Aflatoxins and heroin. BMJ. 299: 492–493. 2507032 10.1136/bmj.299.6697.492 PMC1837336

[22] Williams JH, Aggarwal D, Jolly PE, Phillips T, Wang JS (2005) Connecting the Dots: Logical and Statistical Connections between Aflatoxin Exposure and HIV/AIDS. Peanut CRSP.

[23] Chen SY, Chen CJ, Tsai WY, Ahsan H, Liu TY, Lin JT, Santella RM (2000) Associations of plasma aflatoxin B1-albumin adduct level with plasma selenium level and genetic polymorphisms of glutathione S-transferase M1 and T1. Nutr Cancer. 38: 179–185. 11525595 10.1207/S15327914NC382_6

[24] Flynn JL, Chan J (2001) Immunology of tuberculosis. Annu Rev Immunol. 19: 93–129. 11244032 10.1146/annurev.immunol.19.1.93

[25] Scanga CA, Mohan VP, Yu K, Joseph H, Tanaka K, Chan J, Flynn JL (2000) Depletion of CD4(+) T cells causes reactivation of murine persistent tuberculosis despite continued expression of interferon gamma and nitric oxide synthase 2. J Exp Med. 192: 347–358. 10934223 10.1084/jem.192.3.347 PMC2193220

[26] WHO: Global Tuberculosis Control, WHO Report 2010. In Book Global Tuberculosis Control, WHO Report 2010 (Editor ed.êds.). City: World Health Organization; 2010.

[27] Woodworth JS, Wu Y, Behar SM (2008) *Mycobacterium tuberculosis*-specific CD8+ T cells require perforin to kill target cells and provide protection in vivo. J Immunol. 181: 8595–8603. 19050279 10.4049/jimmunol.181.12.8595 PMC3133658

[28] Hosmer DW, Lemeshow S, May S: Applied survival analysis: regression modeling of time-to-event data. 2nd edn. Hoboken, N.J.: Wiley-Interscience; 2008.

[29] Charalambous S, Grant AD, Moloi V, Warren R, Day JH, van Heiden P, Hayes RJ, Fielding KL, De Cock KM, Chaisson RE, Churchyard GJ (2008) Contribution of reinfection to recurrent tuberculosis in South African gold miners. Int J Tuberc Lung Dis. 12: 942–948. 18647455

[30] Furlan V, Taburet AM (2001) [Drug interactions with antiretroviral agents]. Therapie. 56: 267–271. 11475806

